# Cryogenic Infrared Action Spectroscopy of [H_2_NCO]^+^ and [H_2_NCS]^+^, Protonated Forms of Interstellar HNCO and HNCS

**DOI:** 10.1021/acs.jpca.5c04708

**Published:** 2025-11-03

**Authors:** Marius Gerlach, Noël René Schneider, Sara Petrić, Hunarpreet Kaur, P. Bryan Changala, Britta Redlich, Sandra Brünken

**Affiliations:** † HFML-FELIX, 6029 Radboud University, Toernooiveld 7, 6525 ED Nijmegen, The Netherlands; ‡ Institute for Molecules and Materials, Radboud University, Heyendaalseweg 135, 6525 AJ Nijmegen, The Netherlands; § Center for Astrophysics, Harvard and Smithsonian, Cambridge, Massachusetts 02138, United States; ∥ Photon Science Division, Deutsches-Elektronen-Synchrotron DESY, Notkestraße 85, 22607 Hamburg, Germany

## Abstract

HNCS, isothiocyanic acid, and HNCO, isocyanic acid, are important molecules in the interstellar medium due to their composition of the essential atoms for organic life and their possible prebiotic role. They are assumed to be formed by dissociative recombination of their protonated versions, [H_2_NCO]^+^ and [H_2_NCS]^+^, where protonation may occur either on the N- or the O/S-atom. Here, we report the investigation of [H_2_NCO]^+^ and [H_2_NCS]^+^ by broadband infrared (IR) spectroscopy in a cryogenic 22-pole ion trap instrument via tag-free leak-out spectroscopy. Infrared radiation in the range of 450–3500 cm^–1^ was provided by the infrared free-electron laser FELIX-2 at HFML–FELIX. We predominantly observe the N-protonated isomers, H_2_NCS^+^ and H_2_NCO^+^, with potentially a small contribution of HNCOH^+^. Five fundamental transitions in the range of 450–3500 cm^–1^ were observed of H_2_NCO^+^ and eight fundamental and overtone/combination bands of H_2_NCS^+^. The transitions are assigned with the help of quantum-chemical calculations using a combination of CCSD­(T)/cc-pCVTZ for anharmonic transition energies and ωB97XD/cc-pVQZ for anharmonic transition intensities. Several modes show a rotational substructure, which is discussed in detail for the ν_5_(*b*_1_) bending vibrational mode of H_2_NCS^+^. The data presented in this paper is the first experimental study investigating the IR spectra of these ions and also the first experimental investigation of [H_2_NCS]^+^. This work provides important reference data for upcoming studies of formation mechanisms of HNCO and HNCS at cryogenic conditions.

## Graphical abstract

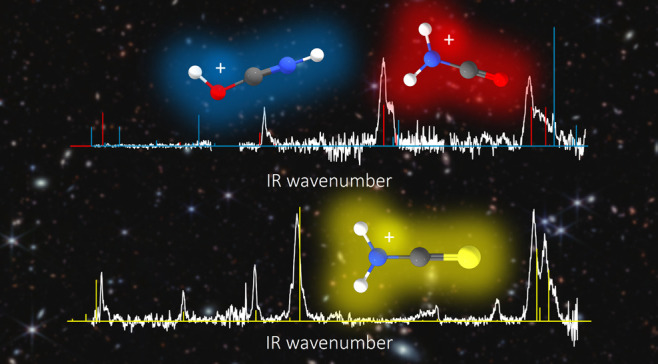

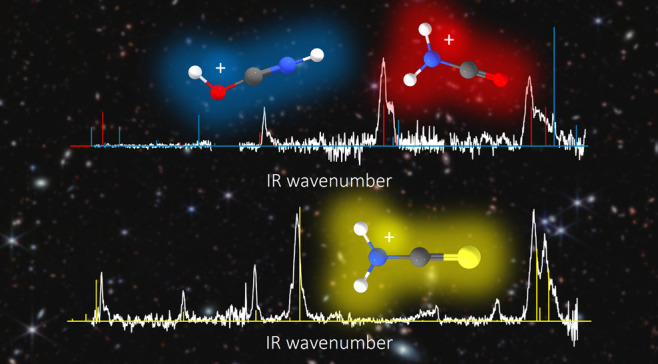

## Introduction

Isocyanic acid, HNCO, is ubiquitous in space. Since its first detection 50 years ago in the galactic center molecular cloud Sagitarrius B2,^1^ it was detected in over 50 sources^2^ including diffuse clouds,^3^ external galaxies,^4–6^ and high- and low-mass star-forming regions.^7–9^ In light of prebiotic chemistry and the origin of life, the potential link between HNCO and formamide, H_2_NCHO, has garnered particular interest. Formamide, which was detected alongside HNCO in various regions,^8,10–15^ is discussed in the literature as a potential precursor for biomolecules. The literature here is vast; as an example, formamide was found to produce the nucleobases thymine,^16^ adenine,^17^ uracil,^17^ cytosine,^18^ and guanine^19^ by metal oxide or mineral catalyzed reactions (see also ref 20 and references therein). The link between HNCO and formamide in the interstellar medium has been investigated using astronomical observations,^10,21^ laboratory experiments,^22–25^ and theoretical investigations.^26^ Recently, Taniguchi et al.^27^ highlighted this link and gave a comprehensive overview of the available literature. The authors ultimately conclude that formamide and HNCO are linked via dual-cyclic H- addition and abstraction reactions involving the carbamoyl radical (H_2_NCO) as an intermediate, as first suggested by Haupa et al. in the ice-phase.^22^ Taniguchi et al. also suggest this as a possible formation pathway of HNCO, where first formamide is produced (from NH_2_ and H_2_CO) and is subsequently dehydrogenated to HNCO.^27^

Isothiocyanic acid, HNCS, has also been detected in space,^28–31^ with a HNCO/HNCS ratio of around 40–60 ^29^ close to the cosmic atomic abundance ratio O/S of 37.^32^ With their recent detection of thiofulminic acid, HCNS, an isomer of HNCS, Cernicharo et al. also modeled the gas-phase chemistry of the CHNO and CHNS isomers.^31^ They found that their models severely underestimate the HNCS abundance indicating the models are either missing formation mechanisms or that the included rate constants are inaccurate.

A search for thioformamide, H_2_NCHS, in Sagittarius B2 based on its rotational spectrum was unsuccessful, with a lower limit for the relative abundance H_2_NCHO/H_2_NCHS of 919,^33^ significantly exceeding the O/S ratio. It seems that, while drawing the analogy between oxygen and sulfur-containing molecules is intriguing, these molecules tend to show different chemical behaviors. The different behavior of sulfur in space is also captured in the sulfur puzzle, which describes the fact that in dense molecular clouds, gas-phase sulfur is depleted by up to 2 orders of magnitude relative to astrochemical models.^34^ This implies that sulfur is locked in a currently unknown reservoir, which makes laboratory experiments on sulfur-containing ions, which may also be found in space, crucial.

Aside from the solid-state reaction mechanism above, HNCO may also be produced in the gas-phase of the interstellar medium by dissociative recombination of the closed-shell protonated counterparts [H_2_NCO]^+^.^37^ Similarly, HNCS may be produced via dissociative recombination of [H_2_NCS]^+^.^36^ The square brackets denote an ambiguous structure since protonation of HNCO and HNCS may occur on either the nitrogen or the oxygen/sulfur leading to, e.g., H_2_NCO^+^ and HNCOH^+^. In both cases, the N-protonated isomers H_2_NCO^+^ and H_2_NCS^+^ are more stable than the O/S-protonated isomers, HNCOH^+^ and HNCSH^+^, by 75 and 38 kJ/mol,^35,36^ respectively. HNCO is postulated to be produced via^37^

1 $$H_{2}+NCO^{+}\to H+HNCO^{+}+42.48\;\mathrm{kJ}/\mathrm{mol}$$

2 $$H_{2}+HNCO^{+}\to H+H_{2}NCO^{+}+91.66\;\mathrm{kJ}/\mathrm{mol}$$

3 $$H_{2}\mathrm{NC}O^{+}+e^{-}\to H+\mathrm{HNCO}$$

The reaction enthalpies above were calculated using the Active Thermochemical Tables.^38^ The gas-phase formation mechanism of HNCS was investigated computationally by Gronowski et al.^36^ They found that H_2_ + HNCS^+^ → H + H_2_NCS^+^ is endothermic with a barrier of 23.7 kJ/mol in contrast to the equivalent reaction 2 for the O-analogue. Instead, they suggest

4 $$NH_{2}+HCS^{+}\to H+H_{2}NCS^{+}+392\;\mathrm{kJ}/\mathrm{mol}$$

5 $$H_{2}\mathrm{NC}S^{+}+e^{-}\to H+\mathrm{HNCS}$$

as a potential formation mechanism for HNCS in the cold environment of space. In the above reactions, the N-protonated isomers were used to compute the reaction energies, although reactions 2 and 4 producing the O/S-protonated isomers are also exothermic and may also lead to HNCO and HNCS by dissociative recombination.^36,37^ Literature on these closed-shell ions is minimal; only rotational spectra of the protonated HNCO isomers H_2_NCO^+^ and HNCOH^+^ have been measured^35^ and were used to detect H_2_NCO^+^ in Sagittarius B2 ^39^ and L483.^40^ Recently, the rotational spectrum of the neutral H_2_NCO radical was measured, but an astronomical search in IRAS 16293–2422 was unsuccessful.^41^ H_2_NCS^+^/HNCSH^+^ were not yet investigated experimentally, and only two computational studies exist focusing on the energetics and formation mechanism of these isomers in space.^36,42^ The permanent dipole moments of H_2_NCO^+^, HNCOH^+^, H_2_NCS^+^, and HNCSH^+^ are calculated to be 4.1,^35^ 2.1,^35^ 3.02,^36^ and 3.03 D,^36^ respectively. Our own calculations for H_2_NCO^+^, HNCOH^+^, and HNCSH^+^ match the literature, while we obtain 3.4 D for H_2_NCS^+^, which appears to better match the trend observed for the O-isomers.

In this paper, we report the generation of [H_2_NCO]^+^ and [H_2_NCS]^+^ and their spectroscopic characterization in the infrared (IR) region from 400 to 3500 cm^–1^ using the broadband tunable infrared light produced by FELIX-2 available at HFML–FELIX. The experimental spectra are assigned using a combination of coupled cluster and DFT calculations. We also discuss the observed rotational substructure.

## Experimental Methods

The experiments were carried out using the FELion 22-pole cryogenic ion trap instrument coupled to the broadly tunable infrared radiation of FELIX-2 at HFML–FELIX.^43^ Here, we will give a brief overview of the setup; a more detailed description is given in previous publications.^44,45^ [H_2_NCO]^+^ and [H_2_NCS]^+^ were produced by ionization of HNCO/HNCS vapor in a Gerlich-type storage ion source (SIS).^46^ HNCS and HNCO samples were synthesized by the addition of 85% phosphoric acid (Sigma-Aldrich) to an aqueous solution of K^+^NCS^–^ (Sigma-Aldrich, ≥99%) and K^+^NCO^–^ (Sigma-Aldrich, ≥97%), respectively.^47,48^ More details about the synthesis are given in the Supporting Information (SI). The samples were stored at –80 °C to avoid decomposition. During experiments, the HNCS sample was held at –20 °C, while the HNCO sample was held at –40 °C using ethanol/dry ice baths. The vapor entered the source chamber through a leak valve, which was regulated to yield a pressure of ∼10^–5^ mbar.

In the SIS, primary ions are produced by electron impact ionization (electron energy 36 eV for HNCS and 40 eV for HNCO) and are trapped in an rf field, allowing secondary reactions between the ions and neutral molecules to occur. Here we commonly observe protonation of neutral precursors,^49^ possibly by the reaction of the neutral precursor gas with H_3_O^+^ or via self-protonation. This is plausible, since the proton affinity of HNCO (protonation at the N-atom) is higher (753 kJ/mol)^50^ than that of H_2_O (691 kJ/mol).^51^ [H_2_NCO]^+^ produced in the Gerlich storage ion source was contaminated by CO_2_ from the HNCO sample and the residual formamide in the experimental chamber from a previous experiment. Thus, we also attempted to produce [H_2_NCO]^+^ by dissociative ionization of neutral formamide vapor at 30 eV electron energy in an electron impact ionization source (EIS). This dissociation channel was already observed in a previous study on the dissociative photoionization of formamide.^52^ Both approaches lead to *m*/*z* = 44. Due to instabilities of FELIX, we were not able to measure the signal at 3400 cm^–1^ when using the EIS. The region from 1270 to 2800 cm^–1^ was measured with both approaches, and a comparison of these spectra is shown in Figure S1 in the SI. In the experimental results shown below, the spectra in the range of 400–1200 and 1400–2700 cm^–1^ were measured from formamide in the EIS, while the data from 2800 to 3700 cm^–1^ were measured using formamide/HNCO in the SIS.

Using an extraction pulse on the source exit lens, the ions produced in the source then enter a quadrupole mass filter, selecting ions with the mass-to-charge ratio of the ion of interest (*m*/*z* = 44 for [H_2_NCO]^+^ and *m*/*z* = 60 for [H_2_NCS]^+^). The selected ions enter the 22-pole ion trap,^44,45^ where they are cooled close to the trap temperature, in this case 18 K, by collision with He gas, pulsed into the ion trap 15 ms before the ions enter the trap. The ions are irradiated with the infrared radiation from FELIX-2 ^43^ at 10 Hz with a pulse energy of up to around a few tens of mJ in the trap region and a FWHM of around 0.6% of the central wavenumber. The photon energy was calibrated using an infrared grating spectrum analyzer. Depending on the settings of FELIX-2, this may lead to uncertainties of around ±3 cm^–1^.^44^ Spectra are recorded using the novel tag-free leak-out action spectroscopic scheme (LOS), first demonstrated for ro-vibrational spectroscopy by Schmid et al.,^53^ and later applied for broadband vibrational spectroscopy using the FELIX FELs by Steenbakkers et al.^54^ Here, the exit voltage is chosen such that the ions are barely trapped, and additional kinetic energy will lead them to leak out of the trap. To promote collisional energy transfer, around ∼10^12^ cm^–3^ of a 1:1 He/Ne mixture is added to the trap. After excitation of a vibration, the vibrating ions can collide with the Ne atoms and convert some of the vibrational energy to kinetic energy, causing them to leave the trap. Following the irradiation, the trap content is extracted, mass-selected by a second quadrupole, and guided toward a Daly detector. The signal is recorded by counting the number of ions that remain after 1.6 s of irradiation. The spectra are normalized to account for varying laser pulse energy and number of laser pulses to calculate the normalized intensity *I* with^44^

6 $$I=-\frac{\ln\left(S/B\right)}{E\cdot N}$$

*E* represents the pulse energy, *N* corresponds to the number of lasers pulses, *S* is the observed ion counts and *B* represents the baseline ion counts. The data are binned using a bin size of 2 cm^–1^. Due to overlapping signals and non-Gaussian peakshapes resulting from underlying rotational substructure, band positions are determined from the position of the signal maximum.

## Computational Methods

To guide and analyze the experiments, quantum chemical calculations were conducted. The geometries of the two lowest lying isomers of protonated isocyanic acid and isothiocyanic acid, H_2_NCO^+^, HNCOH^+^, H_2_NCS^+^, and HNCSH^+^, were optimized using CCSD­(T)/cc-pCVTZ with all electrons active, as implemented in CFOUR.^55^ The N-protonated isomers exhibit *C*_2*v*_ symmetry and a *X* ^1^A_1_ ground state, while O/S protonation leads to *C*_*s*_ symmetry with a *X* A′ ground state. In the next step, harmonic and anharmonic vibrational energies and intensities were computed. The anharmonic calculations used second-order vibrational perturbation theory as implemented in CFOUR. The resulting structures, geometry parameters, and rotational constants are given in the SI in Figure S4 and Tables S1 and S2, respectively. A comparison of the harmonic and anharmonic stick spectra of H_2_NCO^+^, HNCOH^+^, H_2_NCS^+^, and HNCSH^+^ is shown in the SI in Figure S5. No scaling factor is applied to the anharmonic vibrational energies. A few of these anharmonic calculations showed transition intensities for fundamental modes that were at least an order of magnitude larger than that of the harmonic counterpart. This is likely due to close-lying combination or overtone modes leading to resonances, which may erroneously exaggerate the corresponding intensities.^56^ Thus, we also optimized the geometries and calculated anharmonic vibrational energies and intensities using ωB97XD/cc-pVQZ as implemented in the Gaussian16 program package,^57^ which did not show this behavior. A comparison between both approaches is also shown in Figure S6 in the SI. We then combined the transition energies computed by coupled cluster and the transition intensities computed by density functional theory and produced new spectra. These are the calculated spectra that are shown in the Results and Discussion section. The coordinate axis system for the *C*_2*v*_ symmetric H_2_NCS^+^ and H_2_NCO^+^ is chosen according to the recommendations of Mulliken^58^ and IUPAC,^59^ where the molecules lie in the yz-plane and the *x*-axis is the out-of-plane axis (see also the SI). To simulate the rotational fine structure of some of the observed vibrational modes, we used PGOPHER^61^ and the rotational and vibrational constants computed by CCSD­(T)/cc-pCVTZ.

## Results and Discussion

### Mass Spectra

As outlined in the Experimental Methods section, [H_2_NCO]^+^ was produced by the protonation of HNCO and by the dissociative ionization of formamide. [H_2_NCS]^+^ was produced using the protonation of HNCS. The corresponding mass spectra are shown in Figure 1. Formamide in the EIS (Figure 1a) produces *m*/*z* = 44 among many other fragments, most dominantly *m*/*z* = 27 and *m*/*z* = 18, likely corresponding to HCN^+^ and H_2_O^+^, respectively. The mass spectrum of HNCO in the SIS in Figure 1b also shows the production of *m*/*z* = 44, in addition to a very strong signal of protonated formamide at *m*/*z* = 46, which is present from a previous experiment. This means that it is not clear whether *m*/*z* = 44 is produced by protonation of HNCO or by dissociative ionization of formamide. The signal at *m*/*z* = 40 corresponds to Ar, which was used as an inert gas during the storage of the samples.

**Figure 1:**
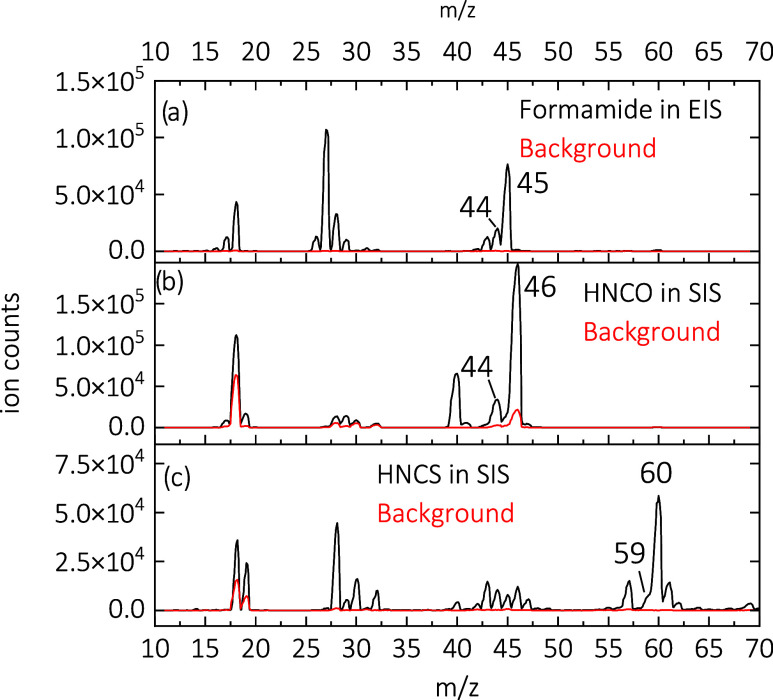
Mass spectra showing the production of [H_2_NCO]^+^ or [H_2_NCS]^+^. (a) Production of [H_2_NCO]^+^ (*m*/*z* = 44) by dissociative ionization of formamide (*m*/*z* = 45) in the electron impact ionization source (EIS). (b) Production of [H_2_NCO]^+^ by protonation of HNCO (*m*/*z* = 43) in the storage ionization source (SIS). (c) Production of [H_2_NCS]^+^ (*m*/*z* = 60) by protonation of HNCS (*m*/*z* = 59) in the SIS.

A typical mass spectrum of [H_2_NCS]^+^ produced via protonation of HNCS in the SIS is shown in Figure 1c. The most intense signal at *m*/*z* = 60 corresponds to protonated HNCS. We also observe a signal at *m*/*z* = 28, likely corresponding to residual N_2_^+^ as well as a number of fragments in the range from *m*/*z* = 42 to 47. Here, *m*/*z* = 44 to 47 may correspond to CS^+^, HCS^+^, NS^+^ and HNS^+^, respectively, while *m*/*z* = 42 and 43 could not be assigned. To record the leak-out spectra and elucidate the structures of [H_2_NCO]^+^ and [H_2_NCS]^+^, the first quadrupole mass filter is tuned to allow *m*/*z* = 44 and *m*/*z* = 60 to enter into the ion trap, respectively.

### Leak-Out Spectroscopy of [H_2_NCO]^+^

The leak-out spectrum of [H_2_NCO]^+^ is shown in Figure 2 (black trace). It consists of three separately scanned regions; see the SI for more details. We observe a very weak signal at 502(3) cm^–1^ and stronger signals at 1542(3), 2336(3) and 3310(4) cm^–1^. The three intense signals show shoulders on the high-energy sides. The anharmonic calculation used CCSD­(T)/cc-pCVTZ and ωB97XD/cc-pVQZ (see Computational Methods section) is shown in Figure 2 in red for H_2_NCO^+^ and in blue for HNCOH^+^. Note that the lower *y*-axis shows the predicted intensities and both calculations are on the same scale.

Based on the comparison of experimental and calculated spectra, we assign the main carrier of *m*/*z* = 44 to be H_2_NCO^+^, although some intensities vary between experiments and theory. The feature around 3310(4) cm^–1^ consists of two vibrations, the H_2_N symmetric and asymmetric stretch vibrations, ν_1_(*a*_1_) and ν_7_(*b*_2_). The calculation predicts both vibrations to be equal in intensity, while we observe a feature for ν_1_(*a*_1_) that is more intense than that for ν_7_(*b*_2_). Additionally, a weak peak is observed at 3464(4) cm^–1^. This may be assigned to the asymmetric stretching mode ν_2_(*a*′) of the opposing H-atoms of the isomer HNCOH^+^, calculated at 3475 cm^–1^. This band is calculated to be the most intense IR transition of HNCOH^+^ and is predicted to be twice as intense as the most intense transitions of H_2_NCO^+^. It should be noted, however, that this assignment is tentative and instead could also originate from ν_7_(*b*_2_) or other combination bands of H_2_NCO^+^. The most intense feature of the spectrum at 2336(3) cm^–1^ consists of a peak and a shoulder on the high-energy side. The peak is assigned to asymmetric NCO stretch ν_2_(*a*_1_). The shoulder could be part of the rotational envelope of the band or another vibrational transition; however, a definitive assignment is not possible. In this region, the first overtone of ν_4_(*a*_1_) of H_2_NCO^+^ and the asymmetric NCO stretch ν_3_(*a*′) of HNCOH^+^ are also predicted. A contribution of HNCOH^+^ can be excluded based on the much lower intensity of this transition compared to the ν_2_(*a*′) mode of HNCOH^+^, which only produced a weak signal. The signal at 1542(3) cm^–1^ is assigned to the H_2_N bending mode ν_3_(*a*_1_) of H_2_NCO^+^. In the region from 400 to 1200 cm^–1^, a weak signal is observed at 502(3) cm^–1^, which is assigned to the H_2_N out-of-plane wagging mode ν_6_(*b*_1_). A plot showing this transition in more detail is included in the SI in Figure S2. This signal shows the largest discrepancy between the predicted and observed intensity and transition energy. All observed vibrational lines and a comparison to the predicted values are summarized in Table 1, including the difference between experiment and theory. Compared to the calculated positions, the observed transitions for the stretching modes are red-shifted by 4 and 12 cm^–1^ for the ν_1_(*a*_1_) and ν_2_(*a*_1_) fundamentals, respectively. The scissoring mode ν_3_(*a*_1_) and the bending mode ν_6_(*b*_1_) are blue-shifted with larger deviations of up to 36 cm^–1^. A study on linear HC_3_O^+^ using similar computational methods showed deviations of at most 11 cm^–1^.^60^ Additionally, some discrepancies are observed between the predicted and the observed intensities, particularly for the ν_7_(*b*_2_) mode at around 3400 cm^–1^ and the ν_6_(*b*_1_) mode at 502(3) cm^–1^. In the case of ν_7_(*b*_2_), no clear band maximum is observed, although it is predicted to be as intense as that of ν_1_(*a*_1_). At these high energies, possible mixing with other vibrational states is possible leading to the observed band shape. In the vicinity, the ν_8_ + ν_2_ combination mode is predicted at 3445 cm^–1^. The signal at 502(3) cm^–1^ is significantly weaker and considerably shifted relative to the predicted ν_6_(*b*_1_) H–N–H out-of-plane wagging mode. It should be noted, however, that ν_6_(*b*_1_) is a c-type transition allowing Δ*K*_a_ = ±1. The rotational constant *A* is calculated at 10.2 cm^–1^ using CCSD­(T)/cc-pCVTZ. This means that the rotational substructure can significantly shift the band maximum by *A*, partly explaining the observed difference. This will be further discussed below for the equivalent mode of H_2_NCS^+^. This may also lead to a broadening of the observed line and, thus, less peak intensity. Another reason for the lower observed intensity may be the leak-out detection scheme. The leak-out rate is dependent on the amount of kinetic energy the ion receives from the collision with the buffer gas to pass the exit barrier. This will lead to less intense signals on the lower energy side of the spectrum, since less vibrational energy is available to be converted to kinetic energy. This may explain the observed difference for the ν_6_(*b*_1_) mode, although as we show below, this is not observed for the low-energy modes of H_2_NCS^+^.

**Figure 2:**
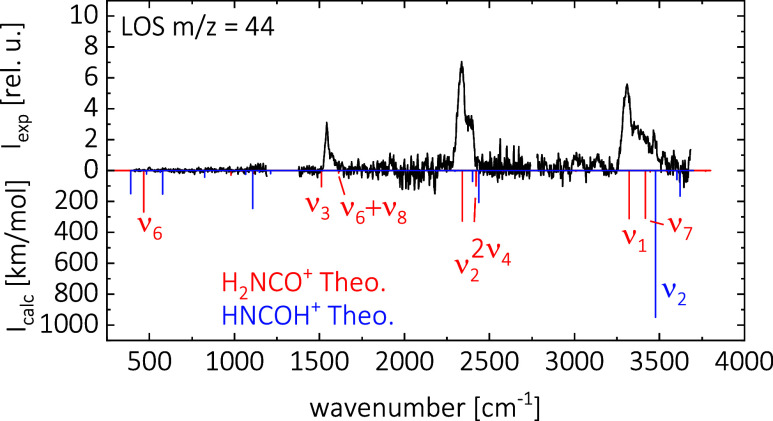
Leak-out spectrum of *m*/*z* = 44 (black) measured at 18 K with Ne as the collision partner. Anharmonic calculations of H_2_NCO^+^ and HNCOH^+^ using CCSD­(T)/cc-pCVTZ and ωB97XD/cc-pVQZ are shown as red and blue sticks, respectively. The symmetry of the vibrations are omitted for clarity.

**Table 1:** Experimental and Calculated Vibrational Data of H_2_NCO^+^ ^a^

mode	description	calc. position [cm^–1^]	calc. int. [km/mol]	exp. position [cm^–1^]	obs – calc [cm^–1^]
ν_1_(*a*_1_)	H–N–H symm. stretch	3322	310	3310(4)	–12
ν_2_(*a*_1_)	N–C–O asymm. stretch	2340	327	2336(3)	–4
ν_3_(*a*_1_)	H–N–H bending	1512	105	1542(3)	+30
ν_4_(*a*_1_)	N–C–O symm. stretch	1214	1	not observed	
ν_5_(*b*_1_)	N–C–O out-of-plane bend	585	<0.5	not observed	
ν_6_(*b*_1_)	H–N–H out-of-plane wagging	466	267	502(3)	+36
ν_7_(*b*_2_)	H–N–H asymm. stretch	3417	309	broad feature	
ν_8_(*b*_2_)	H–N–C in-plane bend	1112	18	not observed	
ν_9_(*b*_2_)	N–C–O in-plane bend	468	1	not observed	

a The experimental positions are determined from the observed signal maximum. The calculated fundamental positions were computed by an anharmonic CCSD­(T)/cc-pCVTZ calculation, and the calculated fundamental intensities using ωB97XD/cc-pVQZ.

In our experiment, we mainly observe the more stable N-protonated isomer H_2_NCO^+^ and possibly a weak contribution of HNCOH^+^. In the case that this signal indeed stems from HNCOH^+^, we can use the observed and computed intensities to give a rough estimate for the H_2_NCO^+^/HNCOH^+^ ratio. Using ν_1_(*a*_1_) of H_2_NCO^+^ with *I*_exp_ = 5.6 and *I*_calc_ = 310 km/mol and ν_2_(*a*′) of HNCOH^+^ with *I*_exp_ = 2.6 and *I*_calc_ = 950 km/mol gives a ratio of 7:1 for H_2_NCO^+^/HNCOH^+^. This calculation assumes that the computed intensities have similar errors and neglects the contribution of underlying vibrational modes to the intensity of both peaks.

### Leak-Out Spectroscopy of [H_2_NCS]^+^

The leak-out spectrum of *m*/*z* = 60 is shown in Figure 3 in black and is compared to the predicted transitions of H_2_NCS^+^, which are plotted in red. Agreement is excellent, so we assign the only carrier of *m*/*z* = 60 to be H_2_NCS^+^. A comparison to the predicted spectrum of HNCSH^+^ is shown in the SI in Figure S3.

**Figure 3:**
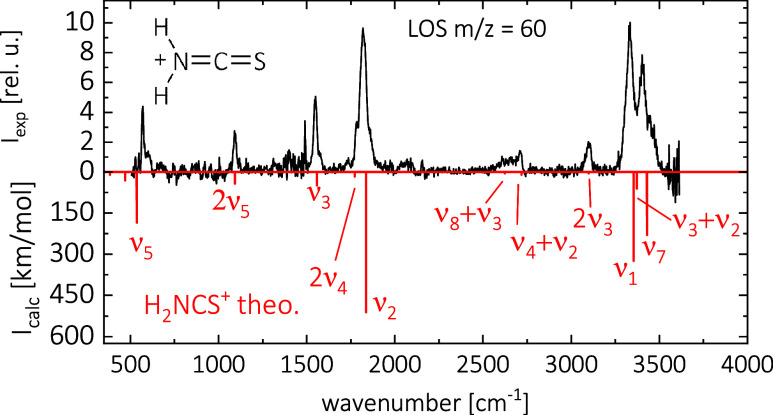
Leak-out spectrum of *m*/*z* = 60 in black measured at 18 K and with Ne as a collision partner. The noisy signal and the spike just below 1500 cm^–1^ are due to the low power of the IR radiation in that region, leading to an amplification of the noise by power normalization. Anharmonic calculation of H_2_NCS^+^ using CCSD­(T)/cc-pCVTZ is shown as red sticks. The symmetry of the vibrations is omitted for clarity.

We observed a total of eight bands. The double peak structure at 3334(4) and 3402(4) cm^–1^ is assigned to the symmetric and asymmetric H_2_N stretching modes ν_1_(*a*_1_) and ν_7_(*b*_2_). At 1818(3) cm^–1^, we observe the asymmetric NCS stretching mode ν_2_(*a*_1_). This peak also features shoulders on the high- and low-energy sides, which may be due to the rotational substructure. The signal at 1550(3) cm^–1^ is assigned to the H_2_N in-plane bending mode ν_3_(*a*_1_). The signals at 570(2) and 1090(3) cm^–1^ correspond to the HNC bending mode ν_5_(*b*_1_) and its overtone, respectively. We can also assign some weaker features to overtones and combination modes. The signal at 3096(4) cm^–1^ originates from the overtone of ν_3_(*a*_1_), and the broad feature at around 2650 cm^–1^ may correspond to the two combination modes ν_8_ + ν_3_ and ν_4_ + ν_2_. The assignment of all observed lines is summarized in Table 2. Again, we also show the differences between experiment and theory. As for H_2_NCO^+^, the deviations are larger than what is observed for HC_3_S^+^ using a similar theory, where deviations of around 15 cm^–1^ are observed.^60^ Possibly, these nonlinear molecules are more difficult to compute accurately with these methods. In the case of H_2_NCS^+^, the intensities, however, match better than those for H_2_NCO^+^, and no large difference in intensity is observed for the lower energy vibrations. One possible explanation is indeed a better coupling of the vibrational energies to the kinetic energy transfer in collisions. The signal intensity is also determined by the relative alignment of the FELIX laser through the trap and the shape of the ion cloud within the trap. It is possible that the spectra of H_2_NCO^+^ in the low wavenumber region were recorded with poor overlap between the laser and the ion cloud leading to a lower signal than should be expected from the predictions. Our normalization procedure, based on the overall laser power through the trap, cannot account for this.

**Table 2:** Experimental and Calculated Vibrational Data of H_2_NCS^+^ ^a^

mode	description	calc. position [cm^–1^]	calc. int. [km/mol]	exp. position [cm^–1^]	obs – calc [cm^–1^]
ν_1_(*a*_1_)	H–N–H symm. stretch	3354	151	3334(4)	–20
ν_2_(*a*_1_)	N–C–S asymm. stretch	1838	442	1818(3)	–20
ν_3_(*a*_1_)	H–N–H bending mode	1557	46	1550(3)	–7
2ν_3_	H–N–H bending mode	3100	5	3096(4)	–4
ν_4_(*a*_1_)	N–C–S symm. stretch	888	<0.5	not observed	
ν_5_(*b*_1_)	H–N–H out-of-plane wagging	536	235	570(2)	+34
2ν_5_	H–N–H out-of-plane wagging	1093	47	1090(3)	–3
ν_6_(*b*_1_)	N–C–S out-of-plane bend	470	5	not observed	
ν_7_(*b*_2_)	H–N–H asymm. stretch	3429	222	3402(4)	–27
ν_8_(*b*_2_)	H–N–C in-plane bend	1078	1	not observed	
ν_9_(*b*_2_)	N–C–S in-plane bend	383	10	not observed	

a The experimental positions are determined from the observed signal maximum. The calculated fundamental positions were computed by an anharmonic CCSD­(T)/cc-pCVTZ calculation and the calculated fundamental intensities using ωB97XD/cc-pVQZ.

The computed energy difference between H_2_NCS^+^ and HNCSH^+^ is lower at 38 kJ/mol^36^ than between H_2_NCO^+^ and HNCOH^+^ at 75 kJ/mol.^35^ The SH-stretching mode ν_2_(*a*′) of HNCSH^+^ is predicted at 3520 cm^–1^ with an intensity of 838 km/mol, placing it within the measured photon range and similar in intensity to the ν_2_(*a*′) of HNCOH^+^. Despite this, no contribution from HNCSH^+^ is observed. This indicates that a thermodynamic argument is not enough to rationalize the product distribution.

### Discussion of Rotational Substructure in ν_5_(*b*_1_) of H_2_NCS^+^

Some of the observed bands show a substructure, likely due to partially resolved rotational transitions. To gain more insights, we attempt to model one of the observed modes using an effective Hamiltonian approach as implemented in the program PGOPHER.^61^ For this, we chose the ν_5_(*b*_1_) mode of H_2_NCS^+^, which is shown in Figure 4 in more detail. We observe a strong signal at 570 cm^–1^, two smaller signals on the low-energy side at 548 and 528 cm^–1^ and a broad feature on the high-energy side. This vibration is a c-type transition meaning the dipole moment changes along the out-of-plane axis, leading to selection rules of Δ*K*_a_ = ±1, Δ*K*_c_ = 0, Δ*J* = 0, ±1.

**Figure 4:**
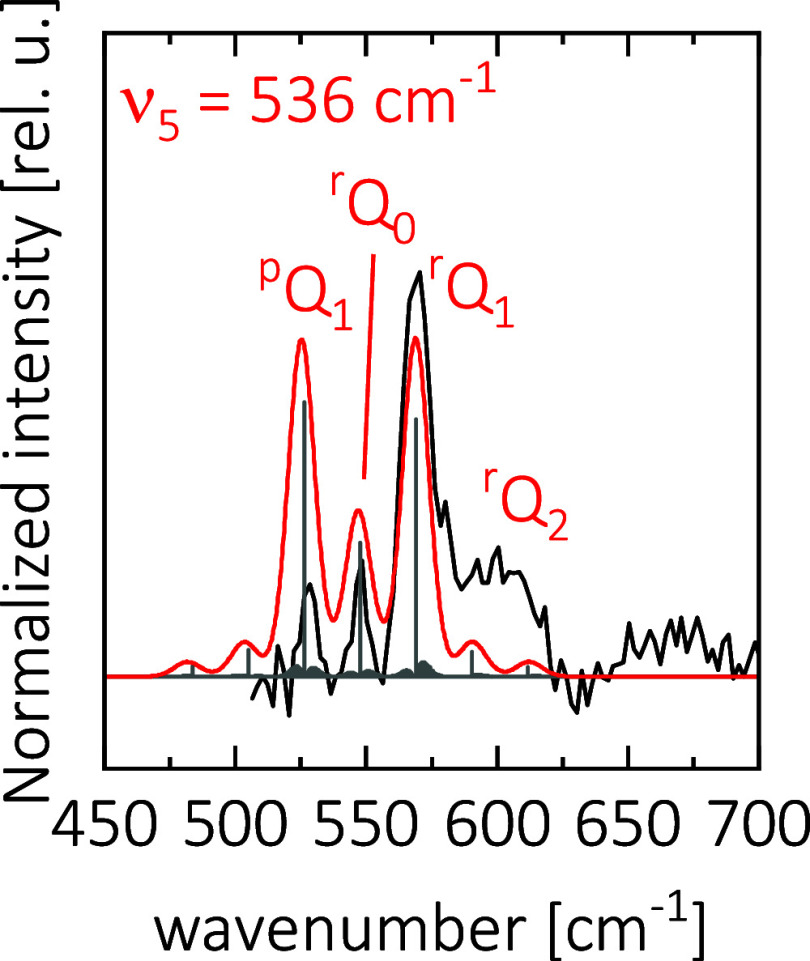
Leak-out spectrum of ν_5_(*b*_1_) of H_2_NCS^+^ in black compared to a simulation using PGOPHER^61^. The simulation consists of the stick spectrum in gray and a Gaussian convolution with a width of 10 cm^–1^. The chosen vibrational origin is 536 cm^–1^, which corresponds to the value calculated by CCSD­(T)/cc-pCVTZ. The simulation is labeled using ^Δ*K*^Δ*J*_*K*a″_. The temperature of the simulation is 40 K. While this simulation temperature is higher than the wall temperature of the trap, this is in line with previous studies using the leak-out scheme.^54,62^

These transitions show a strong ^p,r^Q*_K_*_a_ rotational sub-band structure, each with weak P and R branches. Due to the two equivalent hydrogen atoms, we also expect a 3:1 population ratio between odd and even *K*_a_ levels. This specific vibration is chosen because of the narrower line width of the FEL at low wavenumbers, in this case around 10 cm^–1^ FWHM. Additionally, the c-type transition allows a change in *K*_a_, which will lead to the largest spacing between bands due to the large rotational constant A with a computed value of 10.9 cm^–1^, compared to 0.19 cm^–1^ for B and C. Figure 4 shows a simulation using a Watson’s S-reduced Hamiltonian in the *I* ^r^ representation. H_2_NCS^+^ is a near-prolate asymmetric top with an asymmetry parameter of κ = −0.9994. The rotational parameters used for the simulation were calculated using CCSD­(T)/cc-pCVTZ and are given in the SI Table S3. The transition origin is 536 cm^–1^, which is the value computed by CCSD­(T)/cc-pCVTZ. While the band positions and spacing between experiment and theory match well, the ^P^Q_1_ band is observed to be much weaker than expected. Since this signal is at the edge of the spectral range, the power of FELIX drops off steeply. Typically, we observed around a 1.1 mJ pulse energy at 530 cm^–1^ compared to 1.5 mJ at 570 cm^–1^. This effect should however be compensated by the intensity normalization. Still, this assignment also explains the unexpectedly large difference between the predicted and measured band origin. It is further substantiated by the observation of the 2ν_5_ overtone at 1090(3) cm^–1^, predicted at 1093 cm^–1^. This shows that this vibration is described well by the theory and instead the observed maximum does not correspond to the band origin.

## Conclusion

We report the broadband infrared spectra of H_2_NCS^+^ and H_2_NCO^+^ covering the observable fundamental vibrational transitions in the range from 500 to 3700 cm^–1^. H_2_NCS^+^ ions were generated from protonation of HNCS, while H_2_NCO^+^ was generated by both protonation of HNCO and dissociative ionization of formamide. A total of eight transitions are observed for H_2_NCS^+^, seven of which were assigned to fundamental and overtone transitions. In the case of H_2_NCO^+^, we observed a total of five transitions, which were assigned to four fundamental and one overtone transition. The spectra were recorded using leak-out spectroscopy of the bare ions and were analyzed using high-level CCSD­(T)/cc-pCVTZ as well as ωB97XD/cc-pVQZ calculations. Agreement between experiment and theory is good, though in some cases, the band positions of the vibrational transitions show larger discrepancies than expected. Also, some disagreement is observed between the experimental and predicted intensities. Additionally, a weak contribution of HNCOH^+^ may have been observed at around 3464(4) cm^–1^, with an estimated ratio of 7:1 for H_2_NCO^+^/HNCOH^+^. This can be partially rationalized due to the higher stability of the N-protonated isomer, although kinetic effects also appear to play a role. Despite the lower energy gap between H_2_NCS^+^ and HNCSH^+^, no contribution of HNCSH^+^ is observed. An analysis of the rotational substructure of the low-lying ν_5_(*b*_1_) mode of H_2_NCS^+^ using PGOPHER and calculated rotational constants showed intensity differences between experiment and theory. It also allowed us to explain the large difference between the experimental and predicted vibrational wavenumbers of ν_5_(*b*_1_).

Investigating the kinetics of the reactions that produce these ions will be an important next step in understanding the formation of HNCO and HNCS in space. The kinetics of the subsequent hydrogenations of NCO^+^ to [HNCO]^+^ and [H_2_NCO]^+^ can be studied in the same cryogenic trap setup, similar to previous work on the reaction of the pyridine cation with acetylene.^63^ The IR spectroscopic fingerprints presented in this article will be critical in the identification of the reaction products using leak-out spectroscopy. This will provide us with reaction rates and branching ratios for these reactions, which will be important data points for astrochemical models. On the other hand, the data will also be able to guide high-resolution rotationally resolved vibrational leak-out spectroscopy for example on the features observed in the region above 3000 cm^–1^. This will allow us to investigate H_2_NCS^+^ also with rotational spectroscopy (using LOS double-resonance schemes or Fourier transform microwave spectroscopy), yielding precise rotational constants enabling an astronomical search, an approach that was demonstrated successfully, for example, for H_2_CCCH^+^.^64,65^

## Supplementary Material



## Notes

Comparison of LOS spectra where both the storage ion source with a mixture of HNCO/formamide and the electron impact source with HNCO were used (Figure S1); expanded spectrum of ν_6_(*b*_1_) of H_2_NCO^+^ (Figure S2); comparison of the *m*/*z* = 60 LOS with the stick spectra of H_2_NCS^+^ (red) and HNCSH^+^ (blue) (Figure S3); structures and definition of cartesian coordinate system (Figure S4); geometry parameters of the considered ions (Table S1); rotational parameters of the considered ions computed using anharmonic CCSD­(T)/cc-pCVTZ (Table S2); harmonic and anharmonic predictions for all ions using CCSD­(T)/cc-pCVTZ (Figure S5); comparison of anharmonic vibrational transitions computed using CCSD­(T)/cc-pCVTZ (black) and ωB97XD/cc-pVQZ (red) (Figure S6); constants used in the PGOPHER simulation of the ν_5_(*b*_1_) mode of H_2_NCS^+^ using the Watson S-reduced Hamiltonian (Table S3)^66^ (PDF)
