# Supplementary material for: Cryogenic Infrared Action Spectroscopy of [H2NCO]+ and [H2NCS]+, Protonated Forms of Interstellar HNCO and HNCS
Source: J Phys Chem A. 2025 Nov 3;129(45):10339–47. doi: 10.1021/acs.jpca.5c04708 (PMC12621254; doi:10.1021/acs.jpca.5c04708)
Supplement: Supplementary file 1 [file jp5c04708_si_001.pdf]

Supporting Information for Cryogenic infrared action spectroscopy of $[H_2NCO]^+$ and $[H_2NCS]^+$, protonated forms of interstellar HNCO and HNCS

Marius Gerlach,*,† Noël René Schneider,† Sara Petrić,† Hunarpreet Kaur,† Bryan Changala,‡ Britta Redlich,¶ and Sandra Brünken*,†

†*HFML-FELIX, Toernooiveld 7, 6525ED Nijmegen, the Netherlands; and Institute for Molecules and Materials, Radboud University, Heyendaalseweg 135, 6525 AJ Nijmegen, the Netherlands*

‡*Center for Astrophysics, Harvard and Smithsonian, Cambridge, Massachusetts 02138, United States*

¶*Photon Science Division, Deutsches-Elektronen-Synchrotron DESY, Hamburg, Germany*

E-mail: marius.gerlach@ru.nl; sandra.bruenken@ru.nl

1 Data treatment for the Leak-out spectra of H_2NCO^+

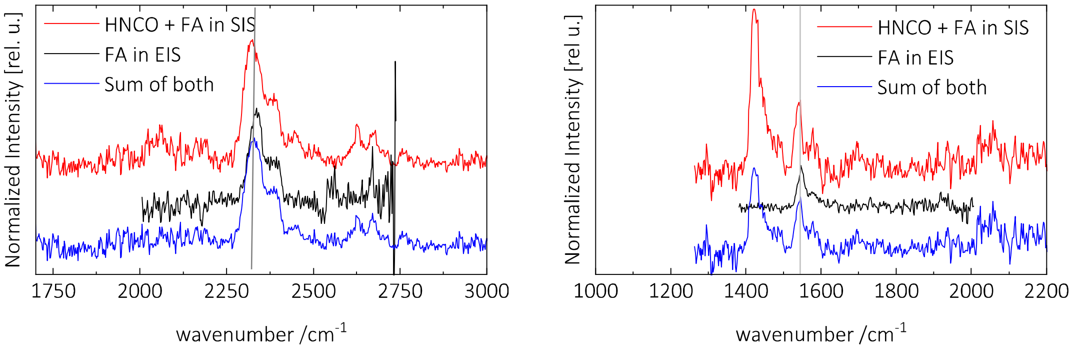

Figure S1: Comparison of LOS spectra where both the storage ion source with a mixture of HNCO/formamide and the electron impact source with HNCO were used.

Figure S1 shows a comparison of the regions where both sources were used. The signals marked with a grey line are due to H_2NCO^+, while the other two signals (1423 and 2657 cm^{-1}) are likely due to contamination. The 1423 cm^{-1} is due to the ν_3 mode of CO_2^+ as reported in the literature.[1] The data shown in the paper for this region is the data set recorded from formamide in the EIS.

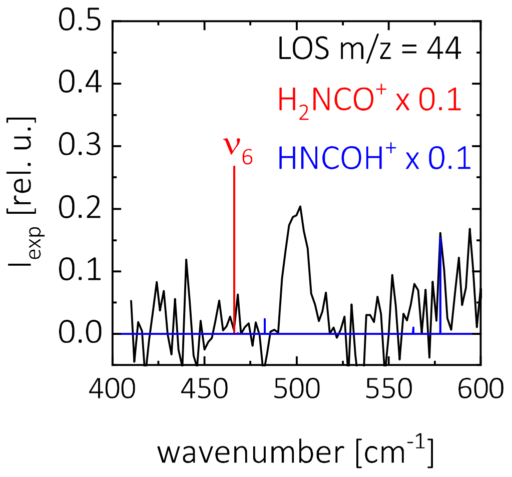

Figure S2: Expanded spectrum of $\nu_6(b_1)$ of H_2NCO^+. Note that the predicted spectra for H_2NCO^+ and $HNCOH^+$ were scaled relative to what is shown in the main paper.

2 Comparison of m/z = 60 data with predicted spectrum of $HNCSH^+$

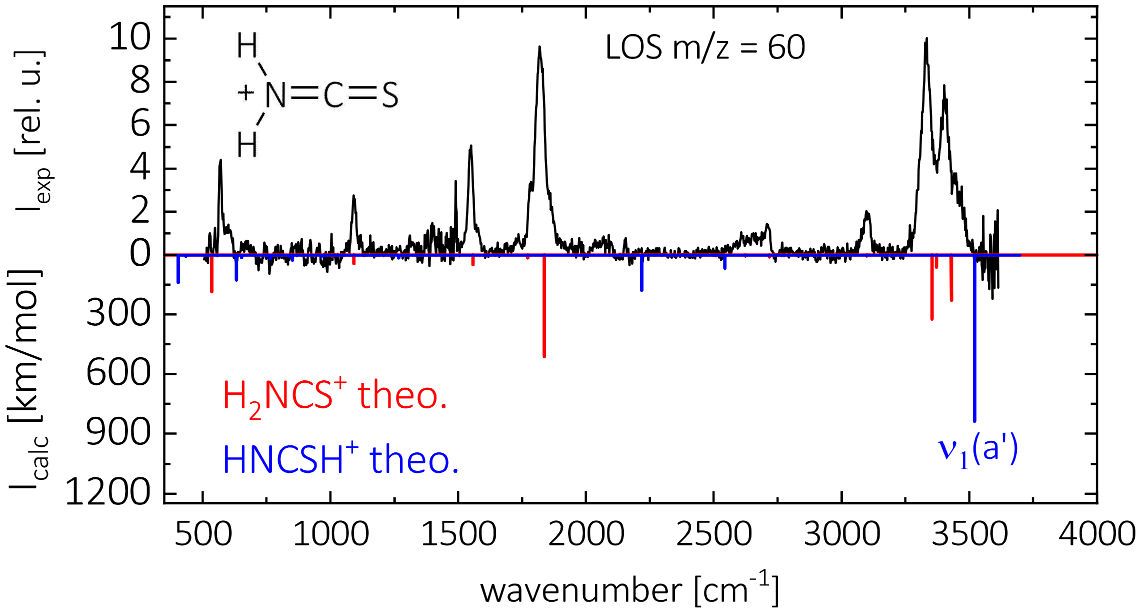

Figure S3: Comparison of the m/z = 60 LOS with the stick spectra of H_2NCS^+ (red) and $HNCSH^+$ (blue).

3 Synthesis of HNCO and HNCS

HNCO and HNCS samples were synthesized by the same method, which has been described for both molecules before.[2,3] The corresponding potassium salt was dissolved in 10 ml of water and cooled using an ice/water mixture. The apparatus was then evacuated and filled with inert gas three times. Over the course of 45 minutes 85% H_3PO_4 was added to the reactions under stirring, while the setup was continuously evacuated. Upon addition of the acid, gas formation can be observed in the reaction mixture. The gaseous reaction products are collected in a cold trap cooled with liquid nitrogen. After the addition is finished, the reaction is allowed to stir for an additional 20 minutes. The raw product of the reaction is purified by exchanging the liquid nitrogen for an ethanol/dry ice cold bath kept at -40°C for HNCO and -30°C for HNCS. The sample containers are then stored at -80°C. During the experiments the concentration of HCN was monitored in the fume hood using a HCN detector. No presence of HCN was observed.

4 Further details on the computational methods

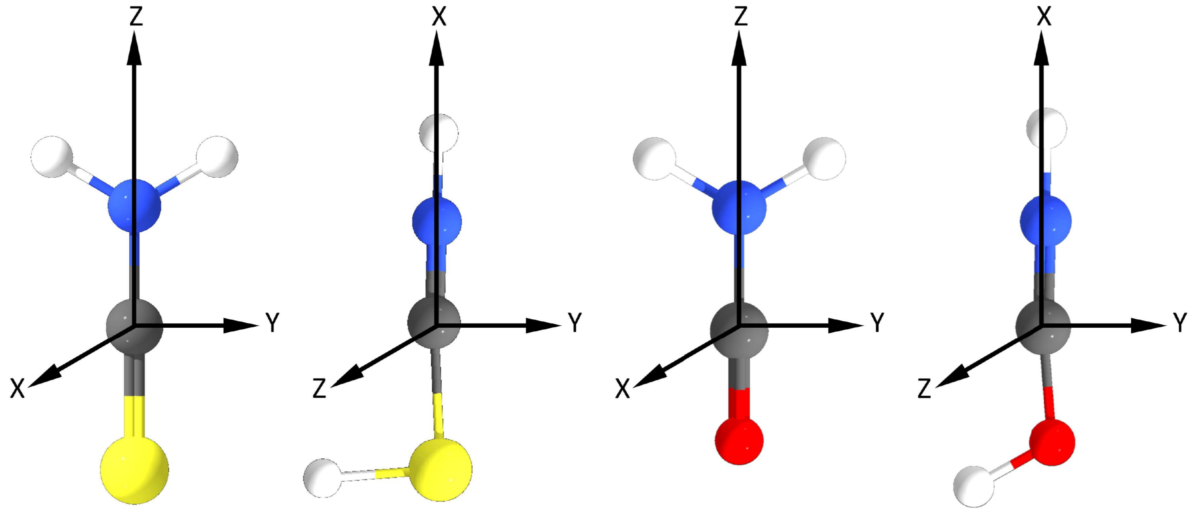

Figure S4: Structures and definition of cartesian coordinate system.

Figure S4 shows the orientation of the cartesian axes of H_2NCS^+, $HNCSH^+$, H_2NCO^+

and $HNCOH^+$. Figure S5 shows a comparison of the harmonic and anharmonic predictions indicating that for H_2NCO^+ and $HNCOH^+$ unexpectedly high intensities were computed for some transitions. Figure S6 shows a comparison between the anharmonic vibrational spectra predicted using CCSD(T)/cc-pCVTZ and ωB97XD/cc-pVQZ.

Table S1: Geometry parameters of the considered ions. Calculated using CCSD(T)/cc-pCVTZ.

Parameter	H_2NCO^+	$HNCOH^+$	H_2NCS^+	$HNCSH^+$
r_{NH}/Å	1.016	1.008	1.014	1.008
r_{NC}/Å	1.274	1.144	1.280	1.150
r_{CX}/Å	1.131	1.250	1.512	1.674
r_{XH}/Å	/	0.977	/	1.348
$\angle HNC$	119.9°	179.1°	120.7°	179.5°
$\angle NCX$	180°	174.2°	180°	175.9°
$\angle CXH$	/	114.9°	/	91.72°

Table S2: Rotational parameters of the considered ions computed using anharmonic CCSD(T)/cc-pCVTZ. Values given in MHz. Experimental values for H_2NCO^+ are given as comparison.[4] Based on the comparison with the experimental values we can estimate the difference between the error of the calculated rotational constants to be of the size of 0.2% to 0.6%.

Parameter	H_2NCO^+	H_2NCO^+ exp.	$HNCOH^+$	H_2NCS^+	$HNCSH^+$
A_e	323101		718565	329439	284534
B_e	10245		9994	5580	5434
C_e	9930		9857	5487	5332
A_0	319071	319782	728630	325909	282028
B_0	10215	10279	9970	5565	5427
C_0	9890	9949	9826	5468	5321
D_K	25.3		445	26.9	17.0
D_{JK}	0.386	0.3775	0.695	0.137	0.123
$10^3 D_J$	2.95	3.0677	2.82	0.923	1.29
$10^6 d_1$	-96	-112	-33	-15	-25
$10^6 d_2$	-26	-38	-7.0	-2.6	-3.1

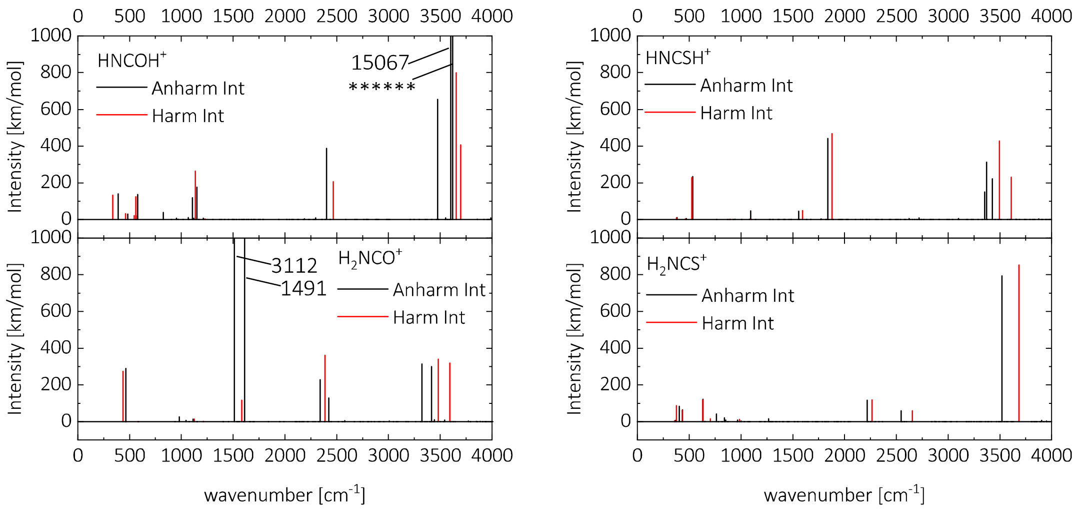

Figure S5: Harmonic and anharmonic predictions for all ions using CCSD(T)/cc-pCVTZ. In cases where the predicted intensity of the anharmonic calculation is significantly higher than that of the harmonic calculation, the transition is labelled with the corresponding intensity in km/mol. ****** represents the intensity output by CFOUR for this transition, likely since the value is too large to be displayed.

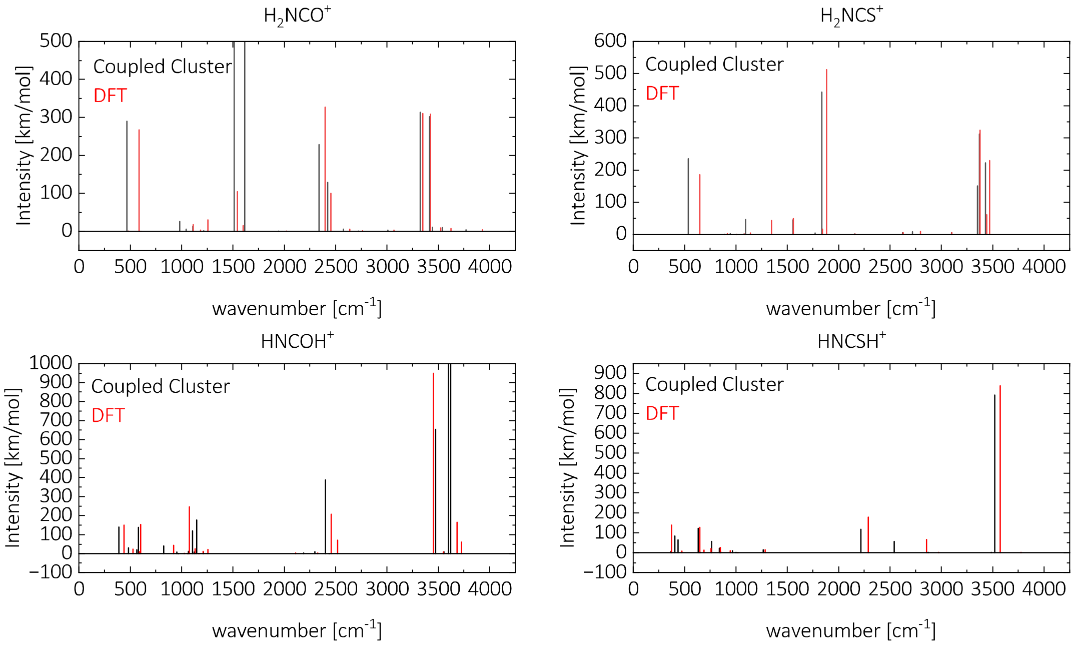

Figure S6: Comparison of anharmonic vibrational transitions computed using CCSD(T)/cc-pCVTZ (black) and ωB97XD/cc-pVQZ (red).

5 PGOPHER simulation

Table S3: Constants used in the PGOPHER simulation of the $\nu_5(b_1)$ mode of H_2NCS^+ using the Watson S-reduced Hamiltonian. Values were computed using CCSD(T)/cc-pCVTZ. All parameters given in the I^r representation. All values given in MHz, except for the band origin, which is given in cm^{-1}.

Parameter	Ground state	$\nu_5(b_1) = 1$
ν_0/cm^{-1}		536
A	325909	325909
B	5565	5565
C	5468	5468
D_K	26.9	26.9
D_{JK}	0.137	0.137
$10^3 D_J$	0.923	0.923
$10^6 d_1$	-15	-15
$10^6 d_2$	-2.6	-2.6

References

(1) Jacox, M. E.; Thompson, W. E. Vibrational spectra of molecular ions isolated in solid neon. 6. Carbon oxide ion (CO4-). *The Journal of Physical Chemistry* **1991**, *95*, 2781–2787, doi: 10.1021/j100160a028.

(2) Ashby, R. A.; Werner, R. L. The vibration-rotation spectrum of HNCO between 1200-450 cm-1. *J. Mol. Spectrosc.* **1965**, *18*, 184–201.

(3) Schaffner, D.; Gerlach, M.; Karaev, E.; Bozek, J.; Fischer, I.; Fink, R. F. Experimental and theoretical investigation of the Auger electron spectra of isothiocyanic acid, HNCS. *Phys. Chem. Chem. Phys.* **2024**, *26*, 27972–27987.

(4) Gupta, H.; Gottlieb, C. A.; Lattanzi, V.; Pearson, J. C.; McCarthy, M. C. Laboratory Measurements and Tentative Astronomical Identification of H_2NCO^+. *Astrophys. J.* **2013**, *778*, L1.
